# Impact of missing electronic fetal monitoring signals on perinatal asphyxia: a multicohort analysis

**DOI:** 10.1038/s41746-025-01665-4

**Published:** 2025-05-01

**Authors:** Debjyoti Karmakar, Lochana Mendis, Emerson Keenan, Marimuthu Palaniswami, Roxanne Hastie, Enes Makalic, Fiona Brownfoot

**Affiliations:** 1 Mercy Hospital for Women/University of Melbourne, Melbourne, VIC Australia; 2 Department of Electrical Engineering, University of Melbourne, Melbourne, VIC Australia; 3 Monash University, Melbourne, VIC Australia; 4 Epworth Freemasons Hospital, Melbourne, Australia

**Keywords:** Translational research, Electrical and electronic engineering

## Abstract

Cardiotocography (CTG) is essential for monitoring high-risk pregnancies, yet perinatal asphyxia prediction accuracy remains limited to 50–55%. Regions of artifacts (missing valid signals)-including signal processing aberrations-possibly contribute to this limitation, highlighted by 40% of FDA reports on intrapartum stillbirths. This cohort study applied causal inference to two digitized CTG databases, analyzing 36,792 labor episodes (>36 weeks) at a tertiary Australian hospital (2010–2021) and externally validating on a Czech dataset (*n* = 552).High rates of missing valid signals (>30% fetal heart rate signal dropout or >1% maternal-fetal heart rate coincidence) was independently associated with asphyxia (aOR 1.47, 95% CI 1.19–1.81); dropout >30% showing stronger link (aOR 1.58, 95% CI 1.13–2.20 Australian dataset; aOR 2.30, 95% CI 1.08–4.91 Czech dataset). Risk of asphyxia increased with higher dropout (>37.45%, aOR 2.21 Australian dataset; >34.01%, aOR 4.08 Czech dataset). Integrating measures of missing valid signals into fetal monitoring algorithms may improve decision-making and neonatal outcomes.

## Introduction

Perinatal asphyxia, a serious neonatal outcome, is caused by impaired maternal-fetal gas exchange. It leads to hypoxia, organ damage, and occasionally death, with up to 40% occurring during labor^1^. It is a leading cause of term newborn morbidity and mortality, responsible for 25% of neonatal deaths, half of third-trimester stillbirths, and one million cases of lifelong disability annually^1,2^. The condition presents substantial public health, emotional, and economic burdens, exemplified by the NHS’s £2.3 billion expenditure on perinatal-related clinical negligence claims in 2019/20^3^.

Cardiotocography (CTG) is a key tool for monitoring fetal well-being in high-risk pregnancies, with clinicians assessing asphyxia risk based on fetal heart rate (FHR) and uterine contraction (tocography) patterns^4^. Since its introduction in the 1960s, CTG has had limited success in reducing perinatal asphyxia but has contributed to a five-fold increase in cesarean section rates^2,5,6^. It records FHR at ~4 Hz using ultrasound (non-invasive) or fetal scalp electrode (ECG, invasive). Proprietary algorithms handle and average signal data to improve visual fidelity by smoothing aberrations such as missing values, signal noise, or maternal heart rates misidentified as FHRs^7–9^. This process forms a continuous graphical trace for clinicians to interpret using predefined rules, though granular numeric details like missing values are masked. These limitations raise concerns about CTG’s accuracy in predicting and preventing perinatal asphyxia. Clinicians detect asphyxia in only 55% of cases, and reliance on visual interpretation of signals may introduce subjective bias, complicating diagnosis^4,5,9–11^. This has driven cesarean rates as high as 50% in some regions utilizing continuous CTG^12,13^. Computerized CTG decision-support tools have also shown limited accuracy (40–50% sensitivity)^2,5,6,10,14^. Missing data and aberrations can account for up to 35% of the total recording length for a patient episode and are often excluded from model training in such computerized machine learning/deep learning model building pipelines^7^. Notably, 40% of FDA reports on intrapartum stillbirths cite concerns about fetal monitoring and maternal-FHR coincidence as contributing factors^15^.

Signal aberrations of the missing valid signal type (also called ‘artifacts’) in CTG, including signal dropout and maternal-FHR coincidence, may arise from technical issues or reflect underlying fetal physiology. Factors such as maternal obesity can increase dropout, while fetal movement may contribute to heart rate confusion. These signal quality issues may be linked to perinatal asphyxia, either as contributors or indicators.

This study examines the association between missing valid signal on CTG and perinatal asphyxia—an area not previously explored. Using a large digital repository of over 400,000 digital CTG episodes, including around 36,000 in labor, we applied causal inference methods to adjust for confounders affecting signal quality and estimated effect sizes for asphyxia risk. Findings were externally validated using a dataset of 552 laboring subjects (Czech CTU-UHB database) following our a priori statistical analysis plan^16^. This approach has the potential to improve neonatal outcomes, particularly in high-risk settings with elevated perinatal asphyxia rates and limited clinical or computational resources by incorporating signal quality-aware models.

## Results

### Association between high missing valid signal in the CTG and fetal asphyxia

**Fig. 1:**
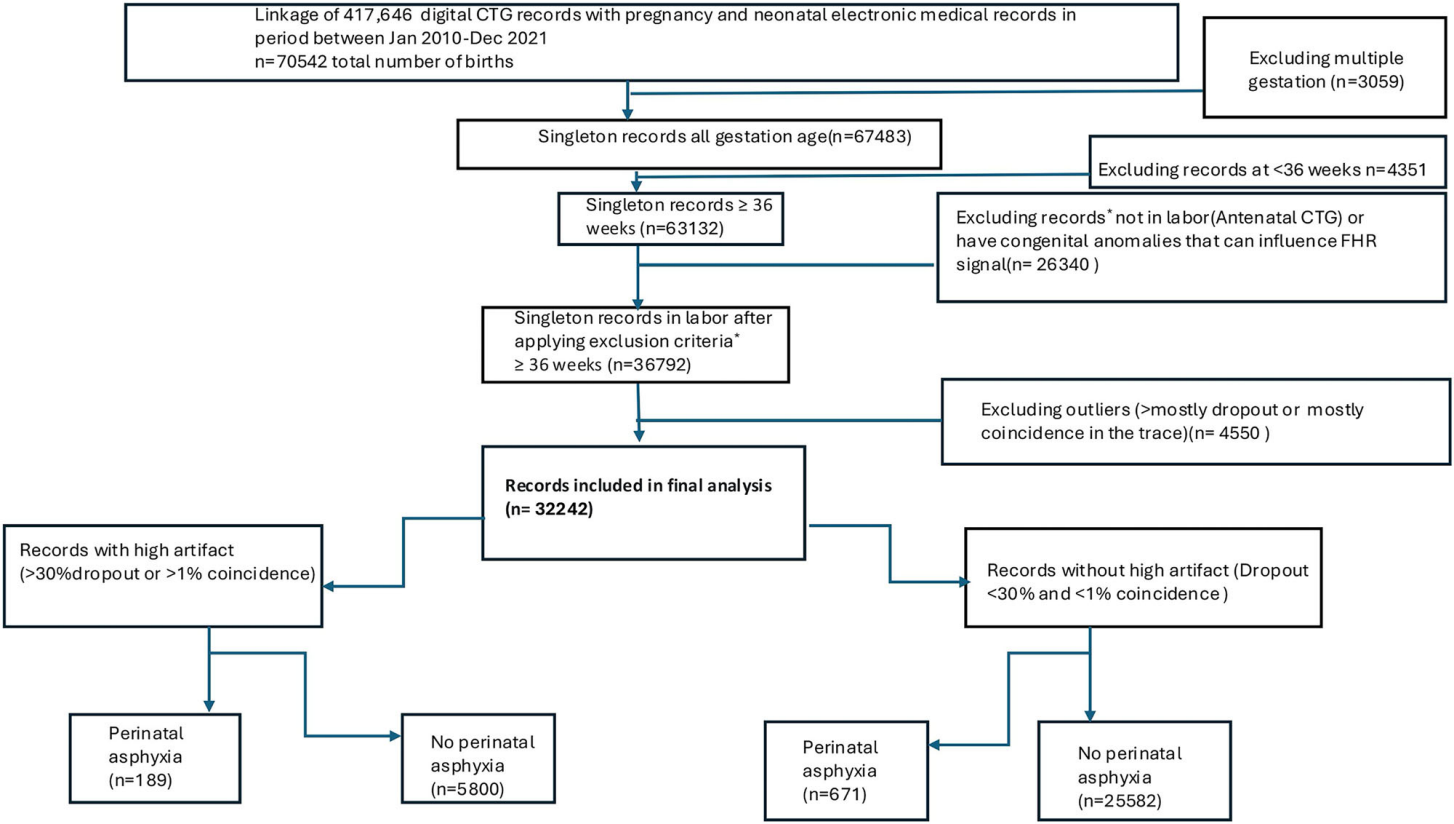
Flow diagram of the study pipeline. The flowchart illustrates the study design, including dataset selection, preprocessing steps, missing valid CTG signal quantification, and analytical framework.

36,792 laboring women at ≥36 weeks with a digitally extracted CTG for the last 60 min before delivery, with a minimum duration of 15 min, from the 1st of Jan 2010 to the 31st of Dec 2021 were screened and 32,242 fit the inclusion criteria. There were 860 cases (2.67%, 95% CI 2.49–2.85) of fetal asphyxia (Fig. 1). Table 1 presents the baseline characteristics of the cohort stratified by exposure to high and not-high missing valid signal on the CTG. There were 26,253 (81.40%) subjects in the non-high missing valid signal group and 5989 (18.60%) in the high missing valid signal group. Maternal age and gestational age were comparable between groups, with a median (IQR) of 32.00 years (29.00–35.00) and 39.40 weeks (38.50–40.30), respectively. Maternal BMI was slightly higher in the high missing valid signal group (24.00 [21.00–28.00]) compared to the non-high missing valid signal group (24.00 [21.00–27.00], *p* < 0.001). Nulliparous women were slightly more represented in the high missing valid signal group (53.98%) compared to the non-high missing valid signal group (52.83%, *p* = 0.11). Notably, a higher proportion of participants in the high missing valid signal group had no identified obstetric risk factors (48.22% vs. 40.58%, *p* < 0.001). Intrapartum events also showed significant differences, with spontaneous onset of labor more common in the high missing valid signal group (55.32% vs. 46.34%, *p* < 0.001) and shorter total labor duration observed in the high missing valid signal group (4.58 h [2.67–7.42] vs. 5.13 h [2.88–8.43], *p* < 0.001).The number of subjects who delivered in the first stage(cesarean section) were 3392 out of 32242(10.52%, 95% CI: 10.19%–10.86%).

**Table 1:** Baseline characteristics stratified by exposure

Characteristic	Missing valid signal (artifact) “Not high” (*n* = 26253)	Missing valid signal (artifact) “High” (*n* = 5989)	Total (*n* = 32242)	*P*-value
Maternal Characteristics				
Age	32.0 (29.0–35.0)	32.0 (29.0–35.0)	32.0 (29.0–35.0)	0.24
BMI	24.0 (21.0–27.0)	24.0 (21.0–28.0)	24.0 (21.0–27.0)	<0.001
* BMI*_*Missing*_	1641 (6.25%)	398 (6.65%)	2039 (6.32%)	
Gestation(weeks)	39.4 (38.5–40.3)	39.4 (38.5–40.3)	39.4 (38.5–40.3)	0.80
Parity				
* Nulliparous*	13870 (52.83%, 52.23–53.44%)	3233 (53.98%, 52.72–55.24%)	17103 (53.05%, 52.50–53.59%)	0.11
* Parous*	12383 (47.17%, 46.56–47.77%)	2756 (46.02%, 44.76–47.28%)	15139 (46.95%, 46.41–47.50%)	
Primary Obstetrics Condition				
* Nil risk factors*	10653 (40.58%, 39.99–41.17%)	2888 (48.22%, 46.96–49.49%)	13541 (42.00%, 41.46–42.54%)	<0.001
* PROM*	3265 (12.44%, 12.04–12.84%)	673 (11.24%, 10.46–12.06%)	3938 (12.21%, 11.86–12.58%)	
* Fetal compromise*^a^	5756 (21.93%, 21.43–22.43%)	1107 (18.48%, 17.52–19.49%)	6863 (21.29%, 20.84–21.74%)	
* Maternal medical conditions*^b^	4684 (17.84%, 17.38–18.31%)	993 (16.58%, 15.66–17.54%)	5677 (17.61%, 17.20–18.03%)	
* Minor obstetrics/medical conditions*	998 (3.80%, 3.58–4.04%)	185 (3.09%, 2.68–3.56%)	1183 (3.67%, 3.47–3.88%)	
* Miscellaneous fetal conditions*^*c*^	897 (3.42%, 3.20–3.64%)	143 (2.39%, 2.03–2.81%)	1040 (3.23%, 3.04–3.42%)	
Labor characteristics/Intrapartum events				
Onset of labor				
* Spontaneous*	12166 (46.34%, 45.74–46.95%)	3313 (55.32%, 54.06–56.57%)	15479 (48.01%, 47.46–48.55%)	<0.001
* Induced*	14087 (53.66%, 53.05–54.26%)	2676 (44.68%, 43.43–45.94%)	16763 (51.99%, 51.45–52.54%)	
Total duration of labor(hours)	5.13 (2.88–8.43)	4.58 (2.67–7.42)	5.03 (2.83–8.25)	<0.001
Duration of first stage of labor (hours)	4.33 (2.33–7.4)	3.83 (2.17–6.5)	4.25 (2.31–7.22)	<0.001
Duration of second stage of labor (hours)	0.45 (0.17–1.18)	0.47 (0.23–1.05)	0.47 (0.17–1.15)	<0.001
Contribution of CTG signal by ultrasound mode	100.00 (17.00–100.00)	78.00 (22.00–100.00)	100.0 (19.00–100.00)	<0.001
Predominant maternal position in labor				
* Dorsal/lithotomy/semi recumbent*	23789 (90.61%, 90.26–90.96%)	5312 (88.70%, 87.87–89.47%)	29101 (90.26%, 89.93–90.58%)	<0.001
* Lateral*	840 (3.20%, 2.99–3.42%)	207 (3.46%, 3.02–3.95%)	1047 (3.25%, 3.06–3.45%)	
* All fours/kneeling*	1324 (5.04%, 4.79–5.31%)	392 (6.55%, 5.95–7.20%)	1716 (5.32%, 5.08–5.57%)	
* Birth stool/squatting/ Standing/water*	300 (1.14%, 1.02–1.28%)	78 (1.30%, 1.04–1.62%)	378 (1.17%, 1.06–1.30%)	
Received regional anesthesia for labor analgesia	12001 (45.71%, 45.11–46.32%)	2138 (35.70%, 34.49–36.92%)	14139 (43.85%, 43.31–44.40%)	<0.001
Presence of meconium-stained liquor	3521 (13.41%, 13.00–13.83%)	816 (13.62%, 12.78–14.52%)	4337 (13.45%, 13.08–13.83%)	0.68
Fetal presentation				
* Non-vertex*	580 (2.21%, 2.04–2.39%)	122 (2.04%, 1.71–2.43%)	702 (2.18%, 2.02–2.34%)	0.44
* Vertex*	25673 (97.79%, 97.61–97.96%)	5867 (97.96%, 97.57–98.29%)	31540 (97.82%, 97.66–97.98%)	
Fetal position (in vertex presentation)				
* Vertex; not occiput anterior*	14768 (57.52%, 56.92–58.13%)	3161 (53.88% 52.60–55.15%)	17929 (56.85%, 56.30–57.39%)	<0.001
* Vertex; occiput anterior*	10905 (42.48% 41.87–43.08%)	2706 (46.12%, 44.85–47.40%)	13611 (43.15%,42.61–43.70%)	
Sentinel events in labor				
* None*	21726 (82.76%, 82.29–83.21%)	4899 (81.80%, 80.80–82.76%)	26625 (82.58%, 82.16–82.99%)	0.10
* Shoulder dystocia, abruption, failed instrumental*	313 (1.19%, 1.07–1.33%)	87 (1.45%, 1.18–1.79%)	400 (1.24%, 1.13–1.37%)	
* Cord accident*	4214 (16.05%, 15.61–16.50%)	1003 (16.75%, 15.82–17.71%)	5217 (16.18%, 15.78–16.59%)	
Mode of birth				
* Vaginal*	17301 (65.90%, 65.33–66.47%)	3671 (61.30%, 60.06–62.52%)	20972 (65.05%, 64.52–65.56%)	<0.001
* Forceps assisted*	3043 (11.59%, 11.21–11.98%)	939 (15.68%, 14.78–16.62%)	3982 (12.35%, 12.00–12.71%)	
* Vacuum assisted*	2187 (8.33%, 8.00–8.67%)	1100 (18.37%, 17.41–19.37%)	3287 (10.19%, 9.87–10.53%)	
* Cesarean section*	3722 (14.18%, 13.76–14.60%)	279 (4.66%, 4.15–5.22%)	4001 (12.41%, 12.05–12.77%)	
Neonatal characteristics				
Birthweight (grams)	3390.0 (3090.0–3700.0)	3350.0 (3050.0–3650.0)	3380.0 (3080.0–3690.0)	<0.001
Gender				
* Female and indeterminate*	12860 (48.98%, 48.38–49.59%)	2889 (48.24%, 46.97–49.50%)	15749 (48.85%, 48.30–49.39%)	0.30
* Male*	13393 (51.02%, 50.41–51.62%)	3100 (51.76%, 50.50–53.03%)	16493 (51.15%, 50.61–51.70%)	
Perinatal asphyxia^d^	671 (2.56%, 2.37–2.75%)	189 (3.16%, 2.74–3.63%)	860 (2.67%, 2.50–2.85%)	0.011

Data are median (IQR) (as not normally distributed), *n* (%, 95% Confidence Interval).

Only BMI had missing data as described.

^a^E.g., Abruption, post-term.

^b^Including hypertensive disease in pregnancy.

^c^E.g., macrosomia, polyhydramnios, unstable lie.

^d^By composite definition.

**Fig. 2:**
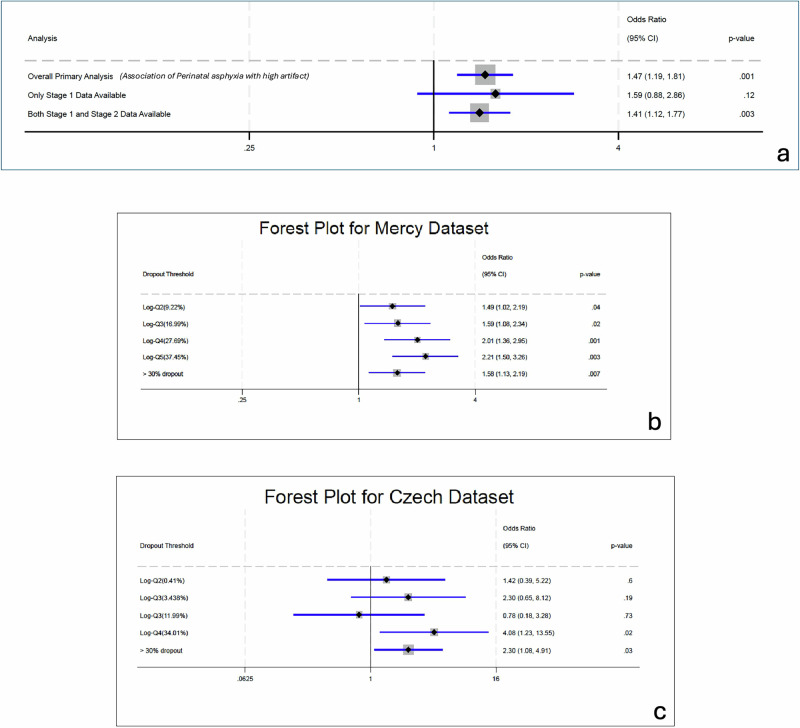
Forest plots showing the association between missing valid signal of CTG and perinatal asphyxia. **a** Odds ratios for perinatal asphyxia in our dataset, including (1) the primary outcome (composite definition of perinatal asphyxia), (2) perinatal asphyxia when only stage 1 labor data is analyzed, and (3) perinatal asphyxia when only stage 2 labor data is analyzed. **b** Odds ratios for asphyxia at increasing thresholds of missing valid CTG signal (specifically signal dropout) in our dataset. **c** Odds ratios for asphyxia at increasing thresholds of missing valid CTG signal (specifically signal dropout) in the validation dataset.

The primary analysis indicates a significant association between high levels of missing valid signals and perinatal asphyxia (Table 2). Unadjusted regression showed an odds ratio (OR) of 1.242 (95% CI: 1.054–1.463; *p* = 0.009). After adjusting for maternal and fetal demographics, clinical features, and obstetric events, the adjusted regression yielded an OR of 1.308 (95% CI, 1.070–1.598; *p* = 0.009). Utilizing doubly robust regression—by combining propensity score matching with regression adjustment to effectively balance the probability of exposure to high levels of missing valid CTG signal and control for confounders—the association of high missing valid signal with perinatal asphyxia strengthened further, resulting in an OR of 1.465 (95% CI: 1.185–1.812; *p* < 0.001) (Fig. 2).

**Table 2:** Association between high missing valid signal on CTG and asphyxia (inverse probability weighted regression analysis)

	Adjusted Odds Ratio (aOR)	95% CI	*p*-value
Overall Primary Analysis- Primary Outcome of Asphyxia^a^			
High missing valid signal and Primary Outcome of Asphyxia	1.47	1.19–1.81	<0.001
Subgroup Analysis by Stage of Labor - Primary Outcome of Asphyxia^a^			
Only Stage 1	1.59	0.88–2.85	0.12
Both stage 1 and stage 2	1.41	1.12–1.77	0.003

1. Odds ratios (ORs) represent the association with asphyxia and were derived using inverse probability weighted regression analysis.

2. No missing data in the missing valid signal and asphyxia labels.

3. Statistical significance set at *p* < 0.05.

4. Doubly robust regression was conducted using Propensity Score Matched and Weighted adjusted regression with inverse probability weighting (IPW).

5. Adjusted ORs are from Complete Case Analysis.

^a^By composite definition.

### Subgroup analysis of primary outcome (Fig. 2, Table 2)

Although our main analysis used only the final 60 min of CTG data prior to birth, we stratified participants based on clinical metadata indicating the presence of timestamps for Stage 1, Stage 2, or both stages, as recorded in mandatory hospital electronic medical record fields. This reflects stage occurrence during the overall labor episode but does not directly compute stage specific contribution of missing valid signal within the analyzed 60-min segment, as fine-grained temporal modeling was beyond the scope of this proof-of-concept study. Based on these metadata-derived indicators, among 32,167 total records, 3392 had Stage 1 only, 75 had Stage 2 only (typically representing expedited or advanced labor), and 28,775 had both stages noted in labor episode. High levels of missing valid signal on CTG were associated with increased odds of perinatal asphyxia in cases where both stages were present (adjusted OR = 1.41, 95% CI: 1.12–1.77; *p* = 0.003). In the Stage 1 only group (*n* = 3392; 22 asphyxia cases), high missing signal was also associated with elevated asphyxia risk (aOR = 1.59, 95% CI: 0.88–2.85), though this did not reach statistical significance (*p* = 0.12). Subgroup analysis was not feasible for the Stage 2 only group due to the small sample size and absence of outcome events.

### Model validation using the Czech dataset

The validation of our doubly robust model on the Czech dataset for the primary outcome of perinatal asphyxia is presented in Fig. 2 and Supplementary Table 8. The association after doubly robust regression with high dropout was an aOR of 2.303 (95% CI: 1.080–4.908; *p* = 0.03) for perinatal asphyxia in the Czech dataset.

### Cross-database association between cumulative signal dropout and perinatal asphyxia

We investigated the association between increasing levels of signal dropout and the risk of perinatal asphyxia in both the Mercy and Czech datasets. Using log-transformed quantiles of dropout amounts (log-Q), the results indicated a progressively stronger association of perinatal asphyxia with increasing dropout levels (Fig. 2, Supplementary Table 8). In both datasets, the highest doubly robust adjusted odds ratios (aORs) for asphyxia were observed in the highest log-transformed quantiles of dropout. In the Mercy dataset, the odds of perinatal asphyxia increased with each successive quintile of dropout, reaching a peak in the highest quintile (aOR 2.21, 95% CI 1.50–3.26, *p* = 0.003 for >37.45% dropout) when compared to the lowest quintile.

Similarly, in the Czech dataset, the highest clinically relevant quintile of dropout was significantly associated with increased odds of perinatal asphyxia (aOR 4.08, 95% CI 1.23–13.55, *p* = 0.02 for dropout greater than 34.01% and below 98.05%) relative to the lowest quartile.

### Statistical validation and data robustness

The significance of dropout association with perinatal asphyxia was confirmed through a Likelihood Ratio (LR) test that compared a model with dropout versus a model without. For our dataset: LR statistic was 56.73, df = 4, *p* < 0.001; for Czech dataset, LR statistic was 27.96, df = 4, *p* < 0.001. For all our doubly robust analyses, effect estimates from the complete case analysis were consistent with those from imputed analyses, indicating missing data did not significantly impact the results (Supplementary Table 9).

## Discussion

This study shows that numeric signal dropout (using 30% dropout as a binary threshold) was associated with perinatal asphyxia in the Mercy (aOR 1.58, 95% CI: 1.13–2.20, *p* = 0.007) and the Czech dataset (aOR 2.30, 95%CI: 1.08–4.91, *p* = 0.03). Furthermore, the effect was cumulative with an aOR 2.21 (95%CI: 1.50–3.26, *p* = 0.003 for >37.45% dropout) in the highest dropout quintile in the Mercy dataset and aOR 4.08 (95% CI 1.23–13.55, *p* = 0.02 for >34.01% dropout) in the Czech dataset. This emphasizes the potential importance of these metrics as early indicators of perinatal asphyxia risk. Our primary aim was to assess the association between signal dropout and perinatal asphyxia using a causal inference framework, rather than to develop a predictive model. However, if used for prediction, then in the primary doubly robust model other clinical variables that contributed included lower birth weight (OR = 0.999, 95% CI: 0.999–0.9998), vertex non-occiput anterior fetal position (OR = 1.37, 95% CI: 1.09–1.73), and increasing gestational age (OR = 1.15, 95% CI: 1.02–1.29).

**Fig. 3:**
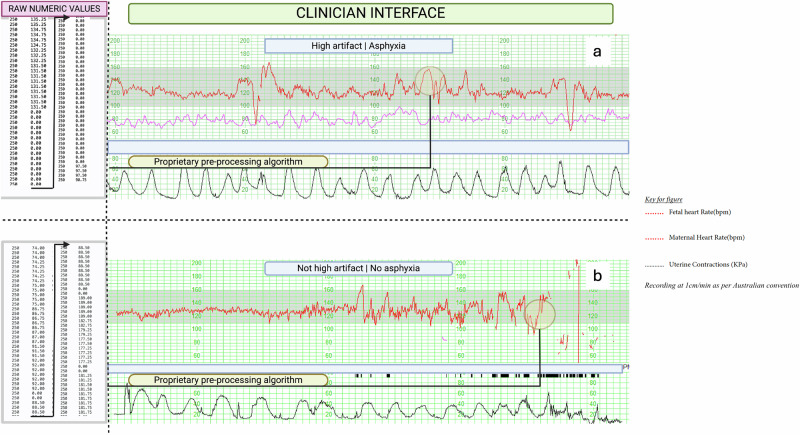
Representation of raw numeric fetal heart rate data and its conversion into a graphical format used by clinicians. Raw fetal heart rate data undergoes proprietary pre-processing to remove outliers and fill in missing values. **a** This recording is a trace with high cumulative signal dropout (>30%), with a representative segment taken from the encircled area. **b** This recording is a trace with low cumulative signal dropout (<30%), with a representative segment derived from the encircled area. Proprietary smoothing in clinician-facing CTG displays may obscure the degree of missing valid CTG signal data. Although the high missing valid signal (**a**, >30% dropout) appears adequate, computational extraction reveals significant signal loss. Missing valid signal (artifact) was quantified as described in the methods section, using a cumulative measure rather than isolated instances. The recording speed is 1 cm/min, per Australian convention. The visual trace is from the Philips Intellispace system, with raw numeric data extracted by our research team in collaboration with Philips.

Figure 3 highlights how missing valid signal on CTG may appear subtle or intermittent in standard CTG displays due to real-time smoothing but can be quantified objectively using raw, unsmoothed data. While longer episodes of signal dropout or maternal-fetal confusion are typically visually detectable, shorter segments, potentially concealed by autocorrelation, may accumulate unnoticed over time. Objective quantification of these segments of missing valid signals could prompt earlier clinical interventions, potentially reducing delays and improving monitoring accuracy. Our approach provides a metric which could support real-time alerts when the level of missing valid signal accumulates to clinically relevant thresholds, enhancing fetal monitoring precision.

Intrapartum perinatal asphyxia contributes to mortality, morbidity and financial burden^2^. Fetal oxygenation in labor is assessed primarily through FHR monitoring using CTG that can detect perinatal asphyxia 54–64% of the time but often “overcalls” it, with sensitivities and specificities below 50% and a low positive predictive value around 30%, with overall accuracy of 50–55%^2,5,6,11^. These limitations have contributed to an alarming fivefold increase in cesarean section rates since CTG’s introduction, underscoring the need for more effective predictive tools to guide clinical decision-making^13^. Computerized analysis of CTG to predict perinatal asphyxia during labor have been disappointing. Computerized CTG analysis shows moderate sensitivity (~50%) and specificity (~80%), with significant variability depending on the system and dataset^5,17^. A big limitation of such reports is small dataset size. A recent systematic review and meta-analysis shows that compared with visual analysis, the use of computerized analysis of intrapartum CTG did not significantly reduce rates of neonatal metabolic acidosis (RR = 0.72, 95%CI: 0.37–1.40) or obstetric intervention, including mode of delivery, admission to neonatal intensive care unit (NICU), Hypoxic Ischemic Encephalopathy (HIE), and perinatal death^17^. This lack of progress may stem, in part, from the exclusion of CTGs with high levels of missing valid signal (artifacts) data from machine learning (ML) model development, as noted in studies where up to 35% of records were discarded due to poor-quality signals (>30% missing data) prior to model training and validation^7,8,18^. Petroziello et al. reported that poor CTG signal quality negatively impacted their MCNN-based predictive models for asphyxia and suggested adjusting classification thresholds based on signal quality^18^.

Signal quality metrics provide actionable real-time insights into potential physiological disruptions during labor as they might represent periods of fetal asphyxia manifesting as reduced variability or other subtle changes in the heart rate patterns that current CTG decision support algorithms fail to capture. There are reports suggesting that CTG signal quality issues may impact CTG interpretation; however, none have systematically examined their effect on asphyxia diagnosis until now. Kiely et al. reported on FDA investigations on sentinel events and found that 40% of 117 CTG-associated deaths were linked to probable signal coincidence, underlining the importance of such metrics^15^. While many studies highlight CTG signal quality’s importance, none have systematically explored the degree of missing valid signal on CTG recording as an independent feature in cases of or predictive of perinatal asphyxia^5,18^. Our gestation cutoff aligns with existing studies, given the focus on HIE^18^.

In our study, we have found after our causal inference modeling that high signal dropout (binary threshold >30%) and the highest quintile of signal dropout had adjusted odds ratios (ORs) 1.58 and 2.21 for asphyxia. These correspond to Numbers Needed to Treat (NNTs, number of cesareans to prevent one additional case of asphyxia) of 67 and 34, respectively. In a separate study (asphyxia incidence 3.2% in a sample size of 4988)^19^, clinically described “Late Deceleration” had an OR of 3.3 which corresponds to a calculated NNT of 16. Combining signal dropout metrics with “late decelerations” in future studies could enhance asphyxia risk prediction and improve intervention strategies, especially given the differences in asphyxia incidence between our study and the “Late Deceleration” study.

In our separate ongoing work using ensemble deep learning models with clinical and time-series data, we incorporate actual labor stage transition timestamps to enable precise temporal mapping of FHR features. In contrast, this study was designed as a foundational, causal inference–based proof of concept to explore associations cumulative missing valid signal proportion over the analyzed segment and perinatal asphyxia, rather than modeling stage-specific physiological transitions. As such, signal metrics were not stratified by the temporal contribution of each labor stage within the analyzed window. A key challenge in computational CTG analysis is the reliance on pre-processed data, which may limit clinical applicability. While portions of missing signal may be visually appreciable, clinicians may not perceive the cumulative burden of signal dropout over time, potentially delaying probe adjustment or increased vigilance. We propose incorporating a cumulative missing valid signal metric on the CTG interface as a first-pass signal integrity alert, updated at regular intervals (e.g., every 15 min). This could enhance clinical awareness of accumulating signal loss, which may otherwise go unnoticed despite being partially visible. Such a feature would embed signal quality monitoring into routine care without adding complexity and could complement second-pass predictive modeling tools for asphyxia risk. Fetal movements, though linked to well-being, are difficult to quantify in causal models and may introduce confounding. While adjusting the ultrasound probe or replacing the ECG electrode is essential, our findings suggest that persistent or cumulatively high levels of missing valid signal may indicate more than technical issues. Backend dropout metrics show that CTG signal data may go amiss even without obvious probe displacement, suggesting potential fetal or placental contributions. Rather than being purely a technical artifact, persistent dropout could serve as an early indicator of fetal compromise. Our research group is investigating the physiology of FHR signals and signal dropout in a sheep model of fetal asphyxia and this is ongoing.

This study provides proof of concept for using real-time signal dropout detection in intrapartum decision support, though the association with asphyxia is not intended to warrant standalone clinical action. These hypothesis-generating findings support future integration of dropout metrics into predictive models. The study’s strength lies in its robust methodology and model development and validation across large and diverse datasets. Our dataset represents one of the largest known repositories of digital CTG recordings linked to clinical outcomes. While previous studies have analyzed CTG data, few have leveraged such a large-scale, structured dataset with validated neonatal outcomes. It may be noted that the odds ratio is higher in the Czech dataset, but the incidence of asphyxia was higher, 7.48% (95% CI 5.42–10.01%) versus 2.67% (95% CI 2.49–2.85%) in the Mercy dataset (z statistic 6.84, *p* < 0.001), and there were significantly fewer covariates available. We applied a predefined causal inference framework to address baseline differences and missing data, using inverse probability-weighted regression to balance clinical and demographic covariates across exposure groups. Covariate balance was confirmed through standardized mean differences and balance plots. To account for potential unmeasured confounding, we performed multiple imputation and sensitivity analyses (Supplementary Table 9). While minor baseline differences—such as BMI and fetal position—were statistically significant due to sample size, absolute differences were small. To mitigate confounding, we implemented a doubly robust approach combining propensity score weighting with outcome regression. Additionally, we examined predictors of high missing valid signal using a directed acyclic graph (DAG)-guided model to account for shared risk factors (Supplementary Information). We restricted analysis to the final 60 min of labor monitoring to align with prior studies and minimize misclassification between antenatal and intrapartum CTGs, ensuring clinical relevance. Decision-to-delivery times for abnormal tracings often fall within this window, supporting its applicability. Our future work will explore forecasting models using longer intrapartum monitoring periods to enhance early detection of asphyxia risk. While our study demonstrates a strong association between missing valid CTG signal and perinatal asphyxia, we acknowledge that causality cannot be definitively established without prospective randomized studies. Evaluating the clinical utility of raw signal data in real-time decision-making will ultimately require larger interventional trials, though such studies are logistically complex and resource-intensive.

In conclusion, our study demonstrates an association between high missing valid CTG signal and perinatal asphyxia, validated across large, diverse datasets. These findings underscore the need for real-time computational algorithms incorporating signal quality as key features to enhance intrapartum asphyxia diagnosis.

## Methods

### Study design and overview (Fig. 1)

Our research question was to determine whether there is an association between the levels of missing valid CTG signal derived from raw backend CTG signal numeric data and perinatal asphyxia in laboring women at ≥36 weeks gestation. A detailed study protocol and a priori statistical analysis plan is included in the Supplementary Information. This study was conducted in three parts; firstly, we explored descriptive statistics of missing valid CTG signal and outcomes. Next, we evaluated the association between high missing valid CTG signal and perinatal asphyxia using causal inference methods, applying adjusted models and propensity score matching to minimize bias and confounding. We then analyzed the impact of increasing signal dropout levels on perinatal outcomes.

### Study population

This study analyzed digital CTG records and outcomes from laboring women at ≥36 weeks gestation over 12 years (Jan 1, 2010–Dec 31, 2021) at a Melbourne tertiary hospital (Fig. 1). We performed computational extraction of raw CTG data from Philips IntelliSpace™ (Philips, Amsterdam, Netherlands) with assistance from Philips. We linked the CTG data to labor episodes, clinical variables, and neonatal outcomes from electronic and physical medical records and the hospital’s morbidity databases. CTG data for analysis was limited to the final 60 min, with a minimum duration of 15 min. This approach, aligned with prior computational fetal monitoring studies^5,18^, optimizes clinical relevance while minimizing confounding from earlier, more variable labor stages. To confirm accuracy, we cross-referenced electronic health record annotations from midwives and obstetricians, ensuring selected tracings corresponded to active labor rather than antenatal recordings. Data was securely stored on the University of Melbourne’s MediaFlux™ system, with computational analysis performed on the Spartan high-performance computing cluster.

For external validation, we used publicly available CTG signals and clinical data from the Czech database. Multiple pregnancies and congenital fetal abnormalities were excluded in both datasets. In both datasets, records with >90% signal dropout were excluded as a predefined quality control measure to ensure data integrity. While signal dropout is a key study variable, extreme dropout levels ( > 90%) likely indicate non-interpretable recordings rather than clinically meaningful signal degradation. This exclusion was applied before outcome analysis to prevent bias and ensure robust, generalizable findings.

### Exposure and outcome

The main exposure was a high level of missing valid signals (artifacts), consisting of FHR signal dropout and maternal-FHR coincidence. FHR “dropout” occurs when the FHR is recorded as zero while active signal acquisition in a live fetus is occurring, while maternal-FHR coincidence (also known as “confusion”) is when FHR and MHR are within five beats per minute (and logged by the monitor backend)^15^. These were measured cumulatively over the full recording window. It should be noted that, in addition, proprietary signal processing algorithms improve trace clarity by minimizing noise through signal autocorrelation techniques. Although this aids in the visual assessment of FHR and uterine activity patterns, short or recurrent interruptions may not be distinctly identified as signal dropout, which could reduce clinicians’ visual recognition of underlying data quality. By Delphi consensus within our multidisciplinary research group (clinicians, data scientists, epidemiologist and signal-processing engineers), high levels of missing valid signals were predefined as >30% dropout or >1% coincidence. Figure 3 illustrates how standard CTG interfaces may inadvertently mask perception of missing valid signals.

The primary outcome was perinatal asphyxia that was defined following Delphi consensus and is in alignment with globally defined criteria^14,20,21^. The presence of any of the following identified the fetus as sustained perinatal asphyxia.

1. Stillbirth attributable to perinatal asphyxia.
2. Neonatal death attributable to perinatal asphyxia.
3. Established diagnosis of hypoxic-ischemic encephalopathy (HIE) based on:
  - Brain injury on imaging.
  - Presence of multisystem organ failure consistent with HIE.
  - Received therapeutic cooling.
4. Received neonatal resuscitation at 10 min, followed by admission to NICU.
5. Apgar score of ≤6 at 10 min or Apgar score of ≤4 at 5 min
6. Arterial cord blood pH <7.05
7. Seizures immediately following birth are thought to be due to asphyxia.

Baseline maternal, fetal, and intrapartum characteristics were compared between groups stratified by the presence of high missing valid signal on CTG. Continuous variables were assessed for normality using the Shapiro-Wilk or Kolmogorov-Smirnov test based on sample size. Normally distributed variables were summarized as mean (standard deviation) and compared using the *t*-test; non-normally distributed variables (such as maternal BMI) were summarized as median (interquartile range) and compared using the Mann-Whitney *U* test. Categorical variables were reported as counts and proportions, with 95% confidence intervals calculated using the Wilson method, and compared using the chi-square test.

### Subgroup analyses

This includes the association of high level of missing valid signal with perinatal asphyxia stratified by stage of labor. *First stage of labor* begins with the onset of regular contractions and ends when the cervix is fully dilated to 10 cm. *Second stage of labor* starts once the cervix is fully dilated and ends with the delivery of the baby.

### Sample size

With a 2.74% (95%CI: 2.49–2.84%) prevalence of perinatal asphyxia (including hypoxic ischemic encephalopathy across varying degrees of severity), a sample size of 9757 participants per group was calculated to detect a 30% relative risk increase with 80% power and a 0.05 significance level.

### Missing data

Missing data was explicitly analyzed for each variable. In our dataset, maternal body mass index (BMI) had 2039 missing values out of 32,242 observations (P*missing* 6.32%, 95% CI 6.06–6.66%)^22^. Missing data was imputed using multiple imputation. Sensitivity analysis was done comparing complete case and imputed data.

### CTG interpretation

Clinicians assess CTG by analyzing FHR patterns and uterine contractions (tocography) using established criteria, including baseline rate, variability, accelerations, decelerations, and contraction frequency. Abnormal patterns, such as late decelerations or prolonged bradycardia, may signal fetal asphyxia. Standard CTG displays use proprietary smoothing algorithms to display a line that can be interpreted, and the raw data is not made available (Fig. 3). This study focuses on quantification of missing valid signals rather than rule-based CTG interpretation.

### Data modeling and statistical analysis

Regression modeling was the principal statistical method employed in this study, comprising a “doubly robust” model^23,24^. We generated a propensity score model (PSM) using logistic regression to balance baseline characteristics. The propensity scores were used to adjust for confounders related to likelihood of exposure to high levels of missing valid signal. We applied propensity scores to create an inverse probability-weighted regression adjustment. Covariate balance was assessed using balance plots. This adjustment was incorporated into a multivariable logistic regression model to estimate effect of missing valid signal on perinatal asphyxia risk. Throughout the manuscript, “doubly robust” refers to this combined method of propensity score matching and inverse probability weighted regression adjustment. Both adjusted and unadjusted odds ratios with 95% confidence intervals are reported.

We included demographic, maternal (including intrapartum events) and neonatal outcome covariates, which are listed in the Supplementary Tables 2 and 10. Final covariate selection for each model was guided by DAGs developed at protocol generation (Supplementary Figs. 1 and 2). Analytically driven polynomial and logarithmic transformation terms were introduced, and covariates were modeled as continuous or categorical variables based on their relationship with the exposure and outcome.

### Model validation

Using the open-source Czech database^16^, we validated our primary model (coding missing valid signal as a binary variable) and secondary model (incremental thresholds of signal dropout). The Mercy and Czech datasets differ in their covariates for modeling “high missing valid signal” and “fetal asphyxia.” The Mercy dataset includes detailed clinical and procedural features, such as diverse primary obstetrics diagnoses, maternal BMI, fetal presentation, fetal position, and raw CTG signals with mode-specific acquisition data. In contrast, the Czech dataset, excluding very high-risk pregnancies, focuses on broader obstetrics or medical diagnoses and labor-related details like labor duration and mode of birth, without raw channel-specific data. Both datasets include gestation, mode of birth, and baby weight, but the Czech dataset adopts a simpler approach, emphasizing broader maternal and fetal characteristics. Additionally, it lacks some criteria from composite asphyxia definitions, such as NICU events and Hypoxic-Ischemic Encephalopathy (HIE) imaging characteristics (Supplementary Table 6 has detailed description). Covariate selection for the PSM and IPW models followed the same principles applied to the Mercy dataset as described above and in Supplementary Table 6. Using components in concordance with our composite criteria of perinatal asphyxia, there were 41 (7.48%, 95%CI: 5.42–10.01%) cases out of 548 valid observations (after excluding apparent outliers like our dataset). MHR-FHR coincidence data is not available in the Czech dataset.

Furthermore, we tested incremental exposure thresholds to assess the consistency of the observed associations in both datasets.

All statistical analyses were performed using STATA™ 18.5, Jamovi™ 2.5.4, and Python™ 3.6. Digital data was extracted from Philips Intellispace cloud-based infrastructure and stored at Mediaflux storage solution and Spartan High-Performance Computing facility provided by University of Melbourne.

### Ethical approval and reporting standards

This cohort study was approved by the Mercy Public Hospitals (Victoria) Human Research Ethics Committee (#R2020-077), with a waiver of informed consent granted per Victorian guidelines for observational studies. We have enclosed the Strengthening the Reporting of Observational Studies in Epidemiology *(STROBE) checklist* in the supplementary information to ensure comprehensive and unbiased reporting of our study^25^.

## Supplementary information

Supplementary material

## Data Availability

All individual-level data of the included cohort can be shared upon reasonable request to the corresponding author and completion of data transfer agreement forms.

## Code availability

The Stata and Python codes, and data key for variables, for data preprocessing, statistical analysis, and causal inference modeling is available upon reasonable request from the corresponding author, subject to completion of data transfer agreement forms.
