# Supplementary material for: Impact of missing electronic fetal monitoring signals on perinatal asphyxia: a multicohort analysis
Source: NPJ Digit Med. 2025 May 1;8:233. doi: 10.1038/s41746-025-01665-4 (PMC12045961; doi:10.1038/s41746-025-01665-4)
Supplement: Supplementary file 1 — Supplementary material [file 41746_2025_1665_MOESM1_ESM.pdf]

SUPPLEMENTARY INFORMATION

TABLE OF CONTENTS

LIST OF INVESTIGATORS **4**

STATISTICAL ANALYSIS PLAN **5**

- **BACKGROUND** 5
- **PROJECT OVERVIEW:** 5
- **Definitions:** 5
- **PICO** 8
- **Study variables:** 9
- **Data Sources** 11
- **STATISTICAL ANALYSIS:** 16
- **BIBLIOGRAPHY FOR STATISTICAL ANALYSIS PLAN** 28

CAUSAL DIAGRAMS IN THE STATISTICAL ANALYSIS PLAN **29**

SUPPLEMENTARY TABLES AFTER DATA ANALYSIS **30**

- **Supplementary Table 6: List of Variables Included in the Models** 30
- **Supplementary Table 7: Summary Statistics Table of Raw FHR Dropout Distribution across both datasets** 32
- **Supplementary Table 8: Validation of Our Doubly Robust Model on Czech Dataset for The Primary Outcome at increasing levels of artifact** 33
- **Supplementary Table 9: Association Between High Artifact and Outcome of Asphyxia Using Doubly Robust Regression Models after Multiple Imputation in THE MERCY DATASET** 34
- **Supplementary Table 10 : Exposure Model for High Artifact (Dropout)** 35

STROBE CHECKLIST 37

LIST OF INVESTIGATORS

1. Dr Debjyoti Karmakar
2. Mr Lochana Mendis
3. Dr Emerson Keenan
4. Prof. Marimuthu Palaniswami
5. Dr Roxanne Hastie
6. Prof. Enes Makalic
7. Assoc Prof. Fiona Brownfoot

STATISTICAL ANALYSIS PLAN

(Approved by research team on May 31st 2024)

The association between intrapartum cardiotocography signal artifacts and perinatal asphyxia

BACKGROUND

Since its introduction in the 1960s, Cardiotocography (CTG) has been essential in monitoring high-risk pregnancies. The CTG is an ultrasound-based technology, and sometimes, the signal can drop out or erroneously detect the maternal trace. This study examines whether there is an association between signal artifact and dropout and perinatal asphyxia (hypoxic ischaemic encephalopathy (HIE) and stillbirth). Our team has access to digital CTG records and outcomes, covering 36,792 laboring women at ≥36 weeks gestation over twelve years, to analyse the association of these artifacts and perinatal asphyxia. The incidence of perinatal asphyxia in hospitals in metropolitan Melbourne is around 2.7%.

PROJECT OVERVIEW:

This study will be performed in three parts:

1) Descriptive analysis for the cohort, on signal artifact and outcome
2) To examine whether there is an association between high signal artifact (signal loss of >30% or coincidence of 1% as defined below) and perinatal asphyxia. We will use both an adjusted model and propensity score matching to find the effect sizes and adjust for differences between the groups at baseline.
3) To quantify the amount of cumulative signal artifact on perinatal outcome and determine a threshold that is associated with perinatal asphyxia.

DEFINITIONS:

1. <u>Artifacts:</u>

Artifacts are aberrations in signal acquisition (Kiely et al., 2019; O'Sullivan et al., 2021). They can be:

1.1. Fetal Heart Rate (FHR) "Dropout": The FHR is present but inaccurately recorded as "zero" by the machine, labelled ("red") by the CTG monitor's Cross-channel verification.
1.2. Maternal Heart Rate (MHR) "Coincidence": Instances when MHR is confused with FHR (defined by MHR and FHR readings within 5 beats per minute of each other).

2. *Definition of high level of artifacts*

"High CTG artifacts" are defined based on the following criteria (Ayres-de-Campos et al., 2015; Kiely et al., 2019): A dropout rate exceeding 30%, as indicated by the machine's quality output grading and time series data described above or a coincidence rate of more than 1% between MHR and FHR. With this definition, we label all CTGs into a binary classification of "*high CTG artifact*" versus "*without high CTG artifact*".

3. *Perinatal asphyxia*

We are using composite criteria for perinatal asphyxia as described below. This definition includes an adaptation of elements of the composite perinatal asphyxia score described by the Oxford Big Data group (Brocklehurst et al., 2018; Petrozziello et al., 2019) and the ANZNN (Australia New Zealand Neonatal Network) 2023 consensus. We have established these criteria by thoroughly integrating existing literature and multidisciplinary discussions with respective domain experts. The presence of **any** of the following identified the fetus as sustained perinatal asphyxia:

1.Stillbirth or neonatal death attributable to perinatal asphyxia.

2.Established diagnosis of hypoxic-ischemic encephalopathy (HIE) based on:

(a)Brain injury on imaging ***or*** *(b)Presence of multisystem organ failure consistent with HIE* ***or*** *(c)Received therapeutic cooling.*

3.Received neonatal resuscitation at 10 minutes, followed by admission to neonatal intensive care unit.

4.Apgar score of ≤6 at 10 minutes or Apgar score of ≤4 at 5 minutes

5. Fetal cord pH <7.05
6. Seizures immediately following birth are thought to be due to asphyxia.

PICO

P: The study population includes 36,792 women in labor at ≥36 weeks gestation with a singleton pregnancy at the Mercy Hospital for Women (MHW) from 2010 to 2021 (12 years). Each woman must have at least 15 minutes of digital cardiotocography (CTG) data. We have excluded multiple pregnancies and pregnancies with congenital fetal abnormalities that are likely to impact CTG features independent of hypoxia.

I/E: The exposure is the presence of '*high CTG artifact'* as defined above.

C: The comparison group comprises laboring women '*without high CTG artifact'*, as defined above.

O: There will be one primary outcome: To determine whether there is an association between high signal artifact and perinatal asphyxia (as defined in table 1).

Secondary outcomes: Examine the association of high signal artifact and independent features of perinatal asphyxia:

1) Stillbirth attributable to perinatal asphyxia.
2) Neonatal death attributable to perinatal asphyxia.

3) Established diagnosis of hypoxic-ischemic encephalopathy (HIE) based on brain injury on imaging, or the presence of multisystem organ failure is consistent with HIE, need for therapeutic cooling.
4) Neonatal IPPV at 10 minutes, followed by admission to NICU.
5) Apgar score of ≤6 at 10 minutes
6) Apgar score of ≤4 at 5 minutes
7) Fetal cord pH <7.05
8) Seizures immediately following birth thought to be due to asphyxia.

Subgroup analysis:

1) Stage of labor and association of high signal artifact and perinatal asphyxia
2) Mode of signal acquisition (ultrasound abdominal CTG or electrical CTG by scalp clip) and the association of signal artifact with perinatal asphyxia.
3) Mode of birth (caesarean section or vaginal birth) and the association of signal artifact with perinatal asphyxia.

STUDY VARIABLES:

Demographics and baseline characteristics.

- Maternal Age at delivery
- Parity
- Maternal Country of birth (%) (Caucasian, Asian, Black, Southeast Asian)
- Birth Weight
- Gestation
- BMI (height and weight)

Exposure variables:

- High CTG artifact

Outcome variables:

Primary outcomes

- Perinatal asphyxia

Secondary outcomes:

Perinatal asphyxia analysis

- Stillbirth attributable to perinatal asphyxia.
- Neonatal death attributable to perinatal asphyxia.
- Established diagnosis of hypoxic-ischemic encephalopathy (HIE) based on brain injury on imaging, or the presence of multisystem organ failure is consistent with HIE, need for therapeutic cooling.
- Neonatal IPPV at 10 minutes, followed by admission to NICU.
- Apgar score of ≤6 at 10 minutes
- Apgar score of ≤4 at 5 minutes
- Fetal cord pH <7.05 or base excess <-12
- Seizures immediately following birth thought to be due to asphyxia.

Clinical variables:

- Main ACOG obstetrics diagnosis categories: *Abruptio placentae, APH/PIH, Pre-eclampsia/Premature rupture of membranes /Post-term pregnancy /Maternal medical conditions /Fetal compromise/logistic or psychosocial reasons, pelvic*

instability/Macrosomia/Prev birth trauma/Fetal abn/Unstable lie,polyhydramnios/Prev Obs hx,VBAC/No medical indication

- Position of presenting part (OA/OT/OP)
- Baby Gender
- Meconium-stained liquor
- Cord Prolapse
- Shoulder dystocia
- Failed instrumental
- Main operative delivery indication -fetal distress/others
- Maternal position at time of birth
- Mode of birth *(Vaginal/Forceps/Vacuum/Caesarean)*
- CTG sample duration (hrs)
- USS: ECG mode ratio
- Regional Analgesia
- Presentation *(Vertex/Breech/Transverse/Brow/Face/Oblique/Compound/Cord/other)*

DATA SOURCES

1. **Birthing outcome system (**BOS© a comprehensive digital record system managed by Management Consultants and Technology Services (MCATS)**) and** electronic medical records (Infomedix CPF) for demographic, medical and labor related variables as per above section.

2. **DecisionPoint™ software**

   Neonatal intensive care admissions, including duration, interventions, diagnoses, and outcomes, including criteria listed in Table 1.

3. CTG records (Philips® through their Intellispace (ISP) systems)

CTGs will be obtained through Philips Intellispace systems.

The records are converted into a customised text file, stored securely on the University of Melbourne's Mediaflux™ system. Half a million digital raw CTG files were screened and relevant CTGs linked to the eligible 36,792 women at ≥ 36 weeks gestation with a singleton pregnancy and in labor and their demographic and clinical variables and neonatal outcomes linked. To ensure correct labor episode linkage at clinical interface we will restrict to in labor CTG data up to 60 mins prior to time of birth.

Descriptive analysis

Population characteristics to be described for

a. Total cohort
b. Subjects with high CTG artifact
c. Subjects without high CTG artifact

For normally distributed data, mean and SD will be represented and for skewed distribution medians and IQR will be represented. For categorical data we will represent counts/frequencies and proportions/relative frequency of each category (Table 1).

SUPPLEMENTARY TABLE 1: BASELINE CHARACTERISTICS AND OUTCOME MEASURES (Planned)

**Characteristic**	**Total**	**Subjects with High CTG Artifact**	**Subjects without High CTG Artifact**	**p-value**
Maternal Age (years)				TBD
	Missing Data %	Missing Data %	Missing Data %	
Parity				TBD
	Missing Data %	Missing Data %	Missing Data %	
Maternal Country of birth (%) SACC, 2016				TBD
	Missing Data %	Missing Data %	Missing Data %	
Birth Weight (kg)				TBD
	Missing Data %	Missing Data %	Missing Data %	

Gestation				TBD
(weeks)	Missing Data %	Missing Data %	Missing Data %	
BMI (18.5-25,25-30,35-40,>40)				TBD
	Missing Data %	Missing Data %	Missing Data %	
Main ACOG obstetrics diagnosis				TBD
categories (%) *Abruptio placentae,APH/PIH,Pre-eclampsia/Premature rupture of membranes /Post-term pregnancy /Maternal medical conditions /Fetal compromise/logistic or psycholsocial reasons, , pelvic instability/Macrosomia/Prev birth trauma/Fetal abn/Unstable lie,poly/Prev Obs hx ,VBAC/No medical indication*	Missing Data %	Missing Data %	Missing Data %	
Position of presenting part (%)				TBD
(OA/OP/Breech/Brow/Face/OT/Shoulder)	Missing Data %	Missing Data %	Missing Data %	
Baby Gender (M/F/I)(%)		\		TBD
	Missing Data %	Missing Data %	Missing Data %	
Meconium-stained liquor yes/no(%)				TBD
	Missing Data %	Missing Data %	Missing Data %	
Cord Prolapse yes /no(%)				TBD
	Missing Data %	Missing Data %	Missing Data %	
Shoulder dystocia (%)				TBD

	Missing Data %	Missing Data %	Missing Data %	
Failed instrumental (%)				TBD
	Missing Data %	Missing Data %	Missing Data %	
Main Operative Delivery indication -fetal distress/others (%)				TBD
	Missing Data %	Missing Data %	Missing Data %	
Maternal position at time of birth (%)	%	%	%	TBD
	Missing Data %	Missing Data %	Missing Data %	
Birth Type (%) *(Vaginal/Forceps/Vacuum/Caesarean)*				TBD
	Missing Data %	Missing Data %	Missing Data %	
CTG sample duration (hrs)				TBD
	Missing Data %	Missing Data %	Missing Data %	
USS: ECG mode ratio				TBD
	Missing Data %	Missing Data %	Missing Data %	
Regional Analgesia (%)				TBD
	Missing Data %	Missing Data %	Missing Data %	
Presentation (%) *(Vertex/Breech/Transverse/Brow/Face/Oblique/Compound/Cord/other)*				TBD
	Missing Data %	Missing Data %	Missing Data %	
High CTG Artifact n/N (%)				TBD
	Missing	Missing	Missing	

	Data %	Data %	Data %	
Perinatal asphyxia %	n/N	n/N	n/N	TBD
	Missing Data %	Missing Data %	Missing Data %	

STATISTICAL ANALYSIS:

<u>Null hypothesis</u>

Subjects in labor with CTG exhibiting high CTG artifact and those who do not display high CTG artifact will have the same risk of perinatal asphyxia of the fetus.

Anticipated sample size

We used the *Oxford Big Data* group's power calculation to guide our sample size. Number of women with CTG 'findings of concern' triggering a 'red alert' in their software decision support population, unadjusted for mode/stage is around 10%. Their study was powered to reduce relative risk of perinatal asphyxia diagnosis incidence among women with CTG findings of concern by 50% with a power of 80% and alpha of 0.05.

All women with CTG 'findings of concern':

	Frequency	Percent	Valid Percent	Cumulative Percent
CTG findings of concern	2335	10.4	10.4	100.0
Total number of subjects	22517	100.0	100.0	

Predicted perinatal asphyxia diagnosis among women with CTG 'findings of concern'

		Frequency	Percent	Valid Percent	Cumulative Percent
Valid	0	2300	98.5	98.5	
	1	35	1.5	1.5	100.0
	Total	2335	100.0	100.0	

0= no perinatal asphyxia, 1= perinatal asphyxia

With our population prevalence of 2.7% of perinatal asphyxia (definition above), a sample size of 9,757 participants in each arm (finding of concern i.e "high CTG artifact" versus "not high CTG artifact" levels) should detect a clinically relevant 30% relative increase in risk of asphyxia with our proposed computerised decision support identifying CTG artifact levels of significance at power of 80% and alpha 0.05.

The effect of high signal artifact will be estimated. This will be presented as a risk ratio and risk difference with corresponding 95% confidence intervals.

Regression modelling to find association of CTG signal artifact with perinatal asphyxia will be done as follows:

A. A propensity score model will be developed to balance baseline characteristics between the groups with high and without high CTG artifact, with variables determined by the authorship team and informed by directed acyclic graphs

DAGs (Link). The background characteristics from the two groups will guide the creation of the direct acyclic graphs. One propensity score will be created for the primary outcome of perinatal asphyxia using logistic regression.

B. The effect of signal artifact on perinatal asphyxia risk will be estimated using multivariable logistic/log-binomial regression with inverse probability weighted regression adjustment to control for baseline differences with an odds ratio and corresponding confidence interval for the population odds ratio. We will adjust for covariates determined by authorship team and informed by directed acyclic graphs (DAGs) to address potential confounders(Link). The final covariates are listed in Table 2.

C. Both adjusted and unadjusted odds ratios with 95% confidence intervals will be reported and compared across the models to assess the impact of adjustment strategies. Table 3 represents the primary and secondary analyses.

SUPPLEMENTARY TABLE 2: PLANNED LIST OF VARIABLES TO BE INCLUDED IN THE MODELS

S No	Variables to include in DAG for propensity score model for predictors of high CTG artifact	Variables to include in DAG for final model for outcome asphyxia
1	Primary obstetrics diagnosis	High CTG artifact
2	Maternal BMI	Primary obstetrics diagnosis
3	Fetal Presentation	Maternal BMI

4	Fetal position	Parity
5	Regional anaesthetic	Gestation at birth
6	Duration of labor stage	Labor onset-spont/induced
7	Primary mode of acquisition	Meconium
8	Mode of birth	Presenting part
9	Baby weight	Fetal position
10	Maternal position	Duration of labor stage
11		Mode of birth
12		Sentinel events in labor
13	-	Operative birth for fetal Distress
14	-	Baby Weight
15	-	Regional anaesthetic
16	-	Baby gender
17	-	Primary mode of acquisition
18	-	Maternal position
19	-	Maternal age

Supplementary Table 3: Primary and secondary analyses

Variable	High CTG artifact (n=xx)	Without hig CTG artifact (n=yy)	Relative risk (95% confidence interval)	
			Crude	Adjusted*
**Primary outcomes**				
Perinatal asphyxia	n (%)	n (%)	Xx (xx.x–xx.x), p=0.xxxx	xx.x (xx.x–xx.x), p=0.xxxx
**Secondary outcomes**				
Stillbirth attributable to perinatal asphyxia.	xx.x (xx.x–xx.x),	xx.x (xx.x–xx.x),	x.xx (x.xx–x.xx), p=0.xxxx	x.xx (x.xx–x.xx), p=0.xxxx
Neonatal death attributable to perinatal asphyxia.	N (%)	n (%)	Xx (xx.x–xx.x), p=0.xxxx	Xx (xx.x–xx.x), p=0.xxxx
Established diagnosis of hypoxic-ischemic encephalopathy (HIE) based on brain injury on imaging, or the presence of multisystem organ failure is consistent with HIE, need for therapeutic cooling.	N (%)	n (%)	Xx (xx.x–xx.x), p=0.xxxx	Xx (xx.x–xx.x), p=0.xxxx
Neonatal IPPV at 10 minutes, followed by admission to NICU.	N (%)	n (%)	Xx (xx.x–xx.x), p=0.xxxx	Xx (xx.x–xx.x), p=0.xxxx
Apgar score of ≤6 at 10 minutes	n (%)	n (%)	x.xx (x.xx–x.xx),	x.xx (x.xx–x.xx), p=0.xxxx

			p=0.xxxx	
Apgar score of ≤4 at 5 minutes				
Fetal cord pH <7.05 or base excess <-12	n (%)	n (%)	x.xx (x.xx–x.xx), p=0.xxxx	x.xx (x.xx–x.xx), p=0.xxxx
Seizures immediately following birth thought to be due to asphyxia.	N (%)	n (%)	x.xx (x.xx–x.xx), p=0.xxxx	x.xx (x.xx–x.xx), p=0.xxxx

For primary outcomes, data are presented as number and percent, 95% confidence interval for proportion, and relative risk and absolute risk with 95% CI.

For secondary outcomes, data are presented as median and interquartile range, number and percent, 95% confidence interval for proportion, and relative risk with 95% CI.

*Adjusted for…

SUPPLEMENTARY TABLE 4: PLANNED SUBGROUP ANALYSIS OF DIFFERENT FEATURES OF LABOR AND SIGNAL ACQUISITION, SIGNAL ARTIFACT AND THE ASSOCIATION OF PERINATAL ASPHYXIA

	**Without high CTG artifact**	**High CTG artifact**	**Relative risk (95% confidence interval)**	
**Variable**			**Crude**	**Adjusted***

**Stage 1**	n (%)	n (%)	Xx (xx.x–xx.x),<br>p=0.xxxx	xx.x (xx.x–xx.x),<br>p=0.xxxx
**Stage 2**	n (%)	n (%)	Xx (xx.x–xx.x),<br>p=0.xxxx	xx.x (xx.x–xx.x),<br>p=0.xxxx
**ECG mode**	n (%)	n (%)	Xx (xx.x–xx.x),<br>p=0.xxxx	xx.x (xx.x–xx.x),<br>p=0.xxxx
**USS mode**	n (%)	n (%)	Xx (xx.x–xx.x),<br>p=0.xxxx	xx.x (xx.x–xx.x),<br>p=0.xxxx
**Total duration of CTG**	n (%)	n (%)	Xx (xx.x–xx.x),<br>p=0.xxxx	xx.x (xx.x–xx.x),<br>p=0.xxxx

Data are presented as number and per cent, a 95% confidence interval for proportion, and relative risk with a 95% CI.

*Adjusted for…

CUMULATIVE EFFECT OF ARTIFACTS ON PREDICTION OF PERINATAL ASPHYXIA.

We wish to determine the cumulative effect of signal artifact and whether this can be used to predict fetal asphyxia. We also seek to determine a threshold of signal artifact that corresponds to fetal asphyxia.

We will use machine learning for this:

1) We will look at the actual CTG artifact metric (percentage of whole duration of CTG record) as the exposure dose and calculate odds of asphyxia for incremental bands of CTG signal artifact (eg 10% bands for dropout) using adjusted model and propensity scoring based models as in previous section.
2) Subsequently, we will apply non-linear analytical methods to examine the relationship between the extent of CTG artifact and the occurrence of perinatal asphyxia.
3) Next, we will develop an algorithm designed to identify a clinically meaningful level of signal artifact that consistently predicts the likelihood of perinatal asphyxia.

324 SUPPLEMENTARY TABLE 5:PROPOSED VARIABLES FOR USE IN STATISTICAL ANALYSIS

**Variable**	**Variable name in the data file**	**Variable description/type**
Baseline Demographic and clinical characteristics		
Maternal age at TOB		Continuous
Maternal Country of birth		*1= Nominal-(recoded to SACC 2016 groups)*<br>*2= Oceania and Antarctica*<br>*3= North-West Europe*<br>*4= Southern and Eastern Europe*<br>*5= North Africa and the Middle East*<br>*6= South-East Asia*<br>*7= North-East Asia*<br>*8= Southern and Central Asia*<br>*9= Americas*<br>*10= Sub-Saharan Africa*<br>*11= Supplementary Codes*
Primary Obstetric diagnosis(ACOG)		Nominal<br>1= *Abruptio placentae,APH*<br>2= *PIH,Pre-eclampsia*<br>3= *Premature rupture of membranes*<br>4= *Post-term pregnancy*<br>5= *Maternal medical conditions*<br>6= *Fetal compromise*<br>7= *logistic or psycholsocial reasons,pelvic instability*<br>8= *Macrosomia*<br>9= *Prev birth trauma*<br>10= *Fetal abn*<br>11= *Unstable lie,poly*<br>12= *Prev Obs hx ,VBAC*<br>13= *No medical indication*
Maternal BMI		Categories 18.5-25,25-30,30-35,35-40,>40

Parity		Continuous
Gestation at birth		Continuous
Labor characteristics		
Labor onset		Nominal<br>0=Spontaneous<br>1= Induced
Membranes rupture		Nominal<br>0=Spontaneous<br>1=ARM<br>2=En Caul
Presence of meconium		Nominal<br>0=No<br>1=Yes
Presentation		Nominal<br>1= Vertex<br>2= Breech<br>3= Transverse<br>4= Brow/Face<br>5= Oblique<br>6= Compound<br>7= Cord<br>8= other.
Position of presenting part		Nominal<br>1= OA<br>2= OP<br>3= Breech<br>4= Brow/Face<br>5= OT<br>6= Shoulder
Regional anaesthetic use for labor analgesia		Nominal<br>0=No<br>1=Yes
Duration of the first stage		Continuous

of labor		
Duration of digital CTG recording for the first stage		Continuous
Duration of the second stage of labor		Continuous
Duration of digital CTG recording for the second stage		Continuous
ECG:USS ratio in stage 1		Continuous
ECG:USS ratio in stage 1		Continuous
Sentinel events in labor		Nominal<br>1= Cord prolapse<br>2= Failed instrumental birth<br>3= Abruptio placentae<br>4= Shoulder dystocia
Maternal position at the time of birth		Nominal<br>1= Dorsal/lithotomy/semi-recumbent<br>2= Lateral<br>3= All fours/kneeling<br>4= Birth stool/squatting<br>5= Standing<br>6= Water
Mode of birth(MOB)		Nominal<br>1= Vaginal<br>2= Forceps<br>3= Vacuum<br>4= Cs
If Operative birth(MOB 2,3,4)- primary indication		Nominal<br>1= Fetal distress<br>0= Other
Baby characteristics		
Baby Birth weight		Continuous

Baby gender at birth		Nominal<br>1= Female<br>2= Indeterminate<br>3= Male
Artifact characteristics		
High CTG artifact		Nominal<br>0= Without high CTG artifact<br>1= High CTG artifact<br>Subcategories:<br>0= Without high CTG artifact _ECG<br>1= High CTG artifact_ECG<br>0= Without high CTG artifact _USS<br>1= High CTG artifact_USS<br>0= Without high CTG artifact _Stage1<br>1= High CTG artifact_stage2
Fetal outcome		
Perinatal asphyxia		Nominal<br>0=No<br>1=Yes

BIBLIOGRAPHY FOR STATISTICAL ANALYSIS PLAN

Ayres-de-Campos, D., Spong, C. Y., & Chandraharan, E. (2015). FIGO consensus guidelines on intrapartum fetal monitoring: Cardiotocography. *International Journal of Gynecology & Obstetrics*, *131*(1), 13-24.

Brocklehurst, P., Field, D., Greene, K., Juszczak, E., Kenyon, S., Linsell, L., Mabey, C., Newburn, M., Plachcinski, R., & Quigley, M. (2018). Computerised interpretation of the fetal heart rate during labor: a randomised controlled trial (INFANT). *Health Technology Assessment*, *22*(9).

Kiely, D. J., Oppenheimer, L. W., & Dornan, J. C. (2019). Unrecognized maternal heart rate artifact in cases of perinatal mortality reported to the United States Food and Drug Administration from 2009 to 2019: a critical patient safety issue. *BMC Pregnancy and Childbirth*, *19*, 1-10.

O'Sullivan, M. E., Considine, E. C., O'Riordan, M., Marnane, W. P., Rennie, J., & Boylan, G. B. (2021). Challenges of developing robust AI for intrapartum fetal heart rate monitoring. *Frontiers in Artificial Intelligence*, *4*, 765210.

Petrozziello, A., Redman, C. W., Papageorghiou, A. T., Jordanov, I., & Georgieva, A. (2019). Multimodal convolutional neural networks to detect fetal compromise during labor and delivery. *IEEE Access*, *7*, 112026-112036.

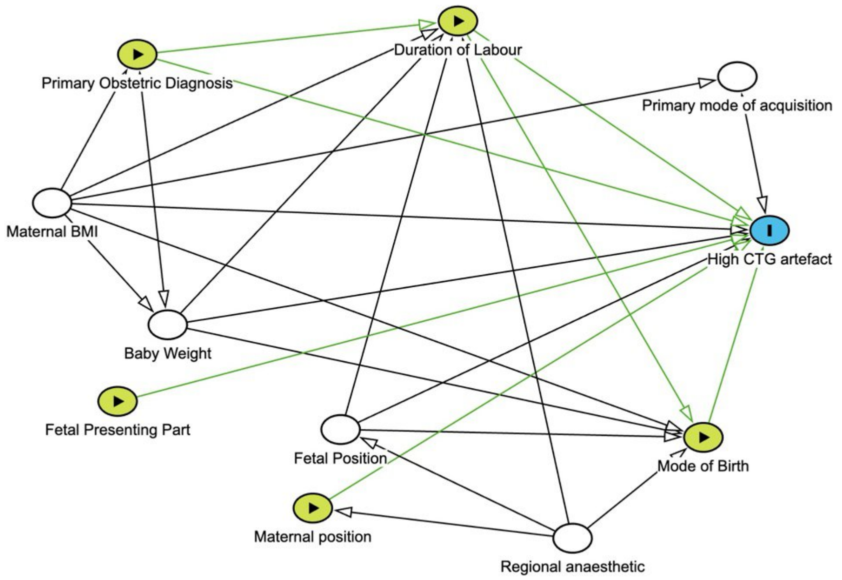

SUPPLEMENTARY FIGURE 1 – CAUSAL DIAGRAM FOR EXPOSURE (HIGH CTG ARTIFACT)

This directed acyclic graph (DAG), created using www.daggity.net, depicts clinical covariates hypothesized to influence the likelihood of high CTG artifact..

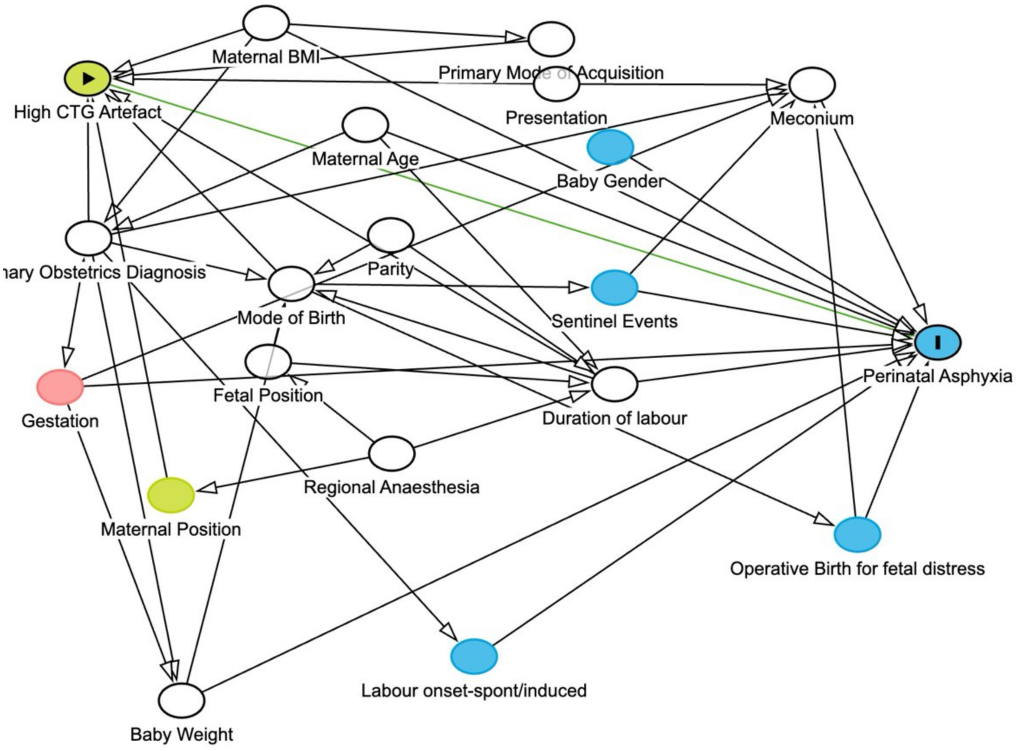

SUPPLEMENTARY FIGURE 2 – CAUSAL DIAGRAM FOR OUTCOME (PERINATAL ASPHYXIA)

This directed acyclic graph (DAG), created using www.daggity.net, depicts clinical covariates hypothesized to influence the likelihood of perinatal asphyxia

SUPPLEMENTARY TABLES AFTER DATA ANALYSIS

Supplementary Table 6: List of Variables Included in the Models

Covariates for propensity score model for exposure “high CTG artefact”	Covariates for inverse propensity weighted adjusted model for outcome “fetal asphyxia”
**Mercy Dataset**	
Primary obstetrics diagnosis	High CTG artefact
Maternal BMI	Primary obstetrics diagnosis
Fetal Presentation	Maternal BMI
Fetal position	Fetal Presentation
Regional anaesthetic	Parity
Duration of labor stage	Gestation at birth
Mode of acquisition	Labor onset-spontaneous/induced
Mode of birth	Meconium
Baby weight	Fetal position
Maternal position	Regional anaesthetic
	Duration of labour stage
	Mode of acquisition
	Sentinel events in labour
	Mode of birth
	Baby weight
	Maternal position
	Baby gender
	Maternal age
**Czech Dataset**	
Mode of acquisition	High CTG Artifact
Duration of labor stage	Mode of acquisition
Obstetrics/medical diagnoses	Duration of labor stage
Fetal presentation	Obstetrics/medical diagnoses
Mode of birth	Fetal presentation
Baby weight	Mode of birth
	Baby weight
	Parity

	Baby gender<br>Maternal age

Supplementary Table 7: Summary Statistics Table of Raw FHR Dropout Distribution across both datasets

	FHR DROPOUT (Mercy)*	FHR DROPOUT (Czech)*
**Mean**	12.71	16.43
**Median**	9.22	6.99
**Standard Deviation (SD)**	13.27	19.98
**Interquartile Range (IQR)**	13.11	27.12
**Log-Q1 upper boundary**	3.19	0.41
**Log-Q2 upper boundary**	9.22	3.43
**Log-Q3 upper boundary**	16.99	11.99
**Log-Q4 upper boundary**	27.69	34.01
**Log-Q5 upper boundary**	37.45	98.05

*The log values were transformed back to the original scale by exponentiation (antilogarithm) using the natural base ‘*e*’.

Supplementary Table 8: Validation of Our Doubly Robust Model on Czech Dataset for The Primary Outcome at increasing levels of artifact

MERCY DATASET				CZECH DATASET			
**Dropout Threshold**	**Odds Ratio (aOR)**	**95% CI**	**p-value**	**Dropout Threshold**	**Odds Ratio (aOR)**	**95% CI**	**p-value**
**Using log transformed dropout quintile* boundaries (Log-Q) and corresponding actual percentage dropout noted in parentheses)**							
Log-Q2(~9.22%)	1.49	1.02-2.19	**0.04**	Log-Q1(.0.41%)	1.42	0.39-5.22	0.60
Log-Q3(~16.99%)	1.59	1.08-2.34	**0.02**	Log-Q2(3.43%)	2.30	0.65-8.12	0.19
Log-Q4(~27.69%)	2.01	1.36-2.95	**<0.001**	Log-Q3(11.99%)	0.78	0.18-3.28	0.73
Log-Q5(~37.45%)	2.21	1.50-3.26	**0.003**	Log-Q4(34.01%)	4.08	1.23-13.55	**0.02**
**Using dropout percentage as binary threshold**							
> 30% dropout	1.58	1.13-2.20	**0.007**	> 30% dropout	2.30	1.08-4.91	**0.03**

Notes:

1. Significance set to <0.05.
2. Doubly robust regression was done using a Propensity Score Matched and Weighted adjusted regression using inverse probability weights.
3. Each log-transformed quantile boundary was used to categorize the population into groups-Q5 was 98.05% in Czech dataset hence modelling not presented as clinically not relevant.
4. All Odds Ratios represented in this table are Doubly robust adjusted ORs

SUPPLEMENTARY TABLE 9: ASSOCIATION BETWEEN HIGH ARTIFACT AND OUTCOME OF ASPHYXIA USING DOUBLY ROBUST REGRESSION MODELS AFTER MULTIPLE IMPUTATION IN THE MERCY DATASET

	Adjusted Odds Ratio (aOR)	95% CI	p-value
**Overall Primary Analysis- Primary Outcome of Asphyxia***			
High Artifact and Primary Outcome of Asphyxia	1.38	1.12-1.70	**0.002**
**Subgroup Analysis Stratified by Mode of Acquisition- Primary Outcome of Asphyxia***			
High Artifact from US Mode	1.25	1.04-1.47	**0.02**
High Artifact from ECG Mode	1.50	1.05-2.13	**0.02**
**Subgroup Analysis Stratified by Modes of Birth- Primary Outcome of Asphyxia***			
Normal birth	1.49	1.13-1.95	**0.004**
Cesarean Section	1.69	1.02-2.83	**0.04**
**Using log transformed dropout quintile* boundaries (Log-Q) and corresponding actual percentage dropout noted in parentheses)**			
Log-Q5(~38.42%)	1.80	1.11-2.91	**0.02**

* **By composite definition**

Notes:

1. There is no missing data in the Artifact and Asphyxia labels.
2. Significance set to <0.05.
3. Doubly robust regression was done using a Propensity Score Matched and Weighted adjusted regression using inverse probability weights.

Supplementary Table 10 : Exposure Model for High Artifact (Dropout)

Variable	Coefficient (b)	Std. Error	p-value	95% Confidence Interval
**log_US_PROP**	-0.908	0.022	<0.001 ***	(-0.952, -0.865)
**log_BMI**	0.908	0.092	<0.001 ***	(0.728, 1.089)
**log_Duration**	-0.129	0.025	<0.001 ***	(-0.177, -0.081)
**Primary Diagnosis (Reference: Normal)**				
PROM	-0.339	0.055	<0.001 ***	(-0.447, -0.231)
Fetal Compromise	-0.388	0.046	<0.001 ***	(-0.479, -0.297)
Maternal Medical Conditions	-0.291	0.048	<0.001 ***	(-0.386, -0.196)
Non-serious	-0.502	0.097	<0.001 ***	(-0.692, -0.313)
Fetal Miscellaneous	-0.362	0.107	0.001 **	(-0.572, -0.151)
**Fetal Presentation**	-0.009	0.113	0.934	(-0.230, 0.211)
**Fetal Position (Reference: Vertex OA)**				
Vertex non-OA	0.022	0.034	0.520	(-0.045, 0.089)
**Maternal Position (Reference: Dorsal/Lithotomy/Semi Recumbent)**				
Lateral	0.148	0.090	0.099	(-0.028, 0.324)
All Fours/Kneeling	0.335	0.068	<0.001 ***	(0.202, 0.468)
Birth Stool/Squatting/Standing/Water	0.085	0.142	0.550	(-0.194, 0.364)
**Regional Anaesthetic**	-0.438	0.040	<0.001 ***	(-0.518, -0.359)
**Mode of Birth (Reference: Vaginal)**				
Forceps	0.222	0.056	<0.001 ***	(0.112, 0.332)
Vacuum	0.483	0.052	<0.001 ***	(0.382, 0.585)
CS	-0.498	0.082	<0.001 ***	(-0.659, -0.338)
**Baby Weight (g)**	-0.000215	0.000038	<0.001 ***	(-0.000290, -0.000141)

Notes:

- ***p < 0.001, *$p < 0.01$, $p < 0.05$

- The model uses a **logistic regression** framework.

STROBE Statement—checklist of items that should be included in reports of observational studies

	Item No.	Recommendation	Page No.	Relevant text from manuscript
**Title and abstract**	1	(*a*) Indicate the study's design with a commonly used term in the title or the abstract	1,2	"This cohort study applied causal inference to two digitized CTG databases" Title "Causal Inference on Electronic Fetal Monitoring Artifacts and Perinatal Asphyxia with International Validation"
		(*b*) Provide in the abstract an informative and balanced summary of what was done and what was found	2,3	The abstract of our manuscript covers this comprehensively.
**Introduction**				
Background/rationale	2	Explain the scientific background and rationale for the investigation being reported	4	Discussed in the *Introduction*, including the significance of CTG and the challenges in predicting perinatal asphyxia.
Objectives	3	State specific objectives, including any prespecified hypotheses	4	Clearly stated in the *Introduction* and *Study Design and Overview* sections.
**Methods**				
Study design	4	Present key elements of study design early in the paper	4,5,6	Presented in the *Methods* section under "Study Design and Overview."
Setting	5	Describe the setting, locations, and relevant dates, including periods of recruitment, exposure, follow-up, and data collection	12,13	Described in *Study Population*, including dates (2010–2021) and the tertiary hospital setting.

Participants	6	(*a*) *Cohort study*—Give the eligibility criteria, and the sources and methods of selection of participants. Describe methods of follow-up<br>*Case-control study*—Give the eligibility criteria, and the sources and methods of case ascertainment and control selection. Give the rationale for the choice of cases and controls<br>*Cross-sectional study*—Give the eligibility criteria, and the sources and methods of selection of participants	12-14	Eligibility criteria and selection methods are detailed in *Study Population*.
		(*b*) *Cohort study*—For matched studies, give matching criteria and number of exposed and unexposed<br>*Case-control study*—For matched studies, give matching criteria and the number of controls per case	12-16	Matching criteria for the Czech dataset validation are described under "Model Validation."
Variables	7	Clearly define all outcomes, exposures, predictors, potential confounders, and effect modifiers. Give diagnostic criteria, if applicable	12-17;Supplementary information	Exposure and outcome definitions are in *Exposure and Outcome*. Direct acyclic graphs included in statistical analysis plan in supplementary material
Data sources/ measurement	8*	For each variable of interest, give sources of data and details of methods of assessment (measurement). Describe comparability of assessment methods if there is more than one group	*12-17; Supplementary information*	Sources and assessment methods are in *Data Modelling and Statistical Analysis*.
Bias	9	Describe any efforts to address potential sources of bias	12-17,7-9	Efforts to minimize bias are described in *Statistical Validation and Data Robustness* and *Missing Data with detailed description of doubly robust regression analysis, sensitivity analysis,likelihood ratio testing.*
Study size	10	Explain how the study size was arrived at	15	Sample size rationale is provided in *Sample Size*.

Continued on next page

Quantitative variables	11	Explain how quantitative variables were handled in the analyses. If applicable, describe which groupings were chosen and why	16,17;Supplementary Material	Handling of quantitative variables is described in *Data Modelling and Statistical Analysis*.
Statistical methods	12	(*a*) Describe all statistical methods, including those used to control for confounding	12-17	Comprehensive methods described in *Data Modelling and Statistical Analysis*.
		(*b*) Describe any methods used to examine subgroups and interactions	12-17	Subgroup analyses are discussed in *Subgroup Analyses*.
		(*c*) Explain how missing data were addressed	15	Missing data treatment is detailed in *Missing Data*.
		(*d*) *Cohort study*—If applicable, explain how loss to follow-up was addressed<br>*Case-control study*—If applicable, explain how matching of cases and controls was addressed<br>*Cross-sectional study*—If applicable, describe analytical methods taking account of sampling strategy		N/A given study premise
		(*e*) Describe any sensitivity analyses	11, supplementary information	Sensitivity analyses mentioned in *Statistical Validation and Data Robustness*.
**Results**				
Participants	13*	(a) Report numbers of individuals at each stage of study—eg numbers potentially eligible, examined for eligibility, confirmed eligible, included in the study, completing follow-up, and analysed	Fig 1, pages 4-6	Numbers at each stage are detailed in *Results*, including exclusions for outliers.<br>16. **Main results:**<br>17. **Other analyses:**
		(b) Give reasons for non-participation at each stage	N/A	
		(c) Consider use of a flow diagram	Fig 1	
Descriptive data	14*	(a) Give characteristics of study participants (eg demographic, clinical, social) and information on exposures and potential confounders		Participant characteristics and confounders are in *Results* (Table

			1).
		(b) Indicate number of participants with missing data for each variable of interest	
		(c) *Cohort study*—Summarise follow-up time (eg, average and total amount)	
Outcome data	15*	*Cohort study*—Report numbers of outcome events or summary measures over time	Numbers and measures of outcomes are reported in *Results*.
		Case-control study—Report numbers in each exposure category, or summary measures of exposure	
		Cross-sectional study—Report numbers of outcome events or summary measures	
Main results	16	(*a*) Give unadjusted estimates and, if applicable, confounder-adjusted estimates and their precision (eg, 95% confidence interval). Make clear which confounders were adjusted for and why they were included	Adjusted and unadjusted estimates are presented with confidence intervals in *Results* (Tables 2 and Supplementary information and figure 2. Supplementary material describe and results summarise which confounders were adjusted for and why they were included
		(*b*) Report category boundaries when continuous variables were categorized	Supplementary tables 7 and 8
		(*c*) If relevant, consider translating estimates of relative risk into absolute risk for a meaningful time period	Described in discussion

Continued on next page

Other analyses	17	Report other analyses done—eg analyses of subgroups and interactions, and sensitivity analyses		Subgroup and sensitivity analyses are included in *Results* and *Subgroup Analyses*.
**Discussion**				
Key results	18	Summarise key results with reference to study objectives	7,8	Summarized in the *Discussion*.
Limitations	19	Discuss limitations of the study, taking into account sources of potential bias or imprecision. Discuss both direction and magnitude of any potential bias	8-10	Discussed under *Discussion*.
Interpretation	20	Give a cautious overall interpretation of results considering objectives, limitations, multiplicity of analyses, results from similar studies, and other relevant evidence	8-11	Provided in *Discussion* with reference to study objectives.
Generalisability	21	Discuss the generalisability (external validity) of the study results	8-12	Discussed in the *Discussion* regarding international implications. Mentioned our validation on external internal dataset.
**Other information**				
Funding	22	Give the source of funding and the role of the funders for the present study and, if applicable, for the original study on which the present article is based	18	Funding details are provided under *Funding*.
